# The Challenge of Melanocytic Lesions in Pediatric Patients: Clinical-Pathological Findings and the Diagnostic Value of PRAME

**DOI:** 10.3389/fonc.2021.688410

**Published:** 2021-06-14

**Authors:** Giuseppina Rosaria Umano, Maria Elena Errico, Vittoria D’Onofrio, Giulia Delehaye, Letizia Trotta, Claudio Spinelli, Silvia Strambi, Renato Franco, Giuseppe D’Abbronzo, Andrea Ronchi, Alfonso Papparella

**Affiliations:** ^1^ Department of Woman, Child and General and Specialized Surgery, University of Campania “Luigi Vanvitelli”, Naples, Italy; ^2^ Department of Pathology, Azienda Ospedaliera di Rilievo Nazionale (AORN) Santobono Pausilipon, Pediatric Hospital, Naples, Italy; ^3^ Pediatric, Adolescent and Young Adults Surgery Division, Department of Surgical, Medical, Pathological, Molecular and Critical Area, University of Pisa, Pisa, Italy; ^4^ Pathology Unit, Department of Mental and Physical Health and Preventive Medicine, University of Campania “Luigi Vanvitelli”, Naples, Italy

**Keywords:** melanoma, children, atypical spitzoid tumor, PRAME, immunohistochemistry

## Abstract

Pediatric melanoma is a rare disease especially in children aged younger than 10 years old. Recent estimates report a rise of disease incidence in both adults and children. Diagnostic work-up is challenging in pediatric melanoma, as it displays a wide range of clinical presentations. Immunohistochemical biomarkers have been reported as predictors of malignancy in melanoma, however data specific to pediatric melanoma are poor. Our study aims to contribute to provide evidence of pediatric melanoma clinical features and differential diagnosis in this patient population. We describe our experience with a retrospective case series of pigmented skin lesions including malignant melanoma, atypical spitzoid tumor, and benign nevi in children and adolescents aged less than 16 years. We described the clinical and demographic characteristics of the cohort and evaluated the immunohistochemical expression of the PReferentially expressed Antigen in MElanoma (PRAME) for differential diagnosis of melanoma in children. The series displayed a similar distribution of melanoma between males and females, and the most common site of melanoma onset were the upper and lower limbs. In our cohort, PRAME was negative in most cases. Focal and slight positivity (from 1 to 5% of the neoplastic cells) was observed in four cases (two Spitz nevi and two atypical Spitz tumors). A moderate positivity in 25% of the neoplastic cells was observed in one case of atypical Spitz tumor. Immunohistochemical expression of PRAME might be useful in the differential diagnosis of malignant melanoma.

## Introduction

Malignant melanoma (MM) affects mainly the adult population, and about 14% of patients aged >18 years-old develop MM during their life according to recent studies (1). Although MM is rare in pediatric age, it is the most common form of skin cancer in children. The incidence increases with age: it is a rare neoplasm in children aged less than 10 years (annual incidence of 0.7–0.8 per million). However, this cancer cannot be considered a rare disease in teenagers, as its incidence is above two cases per million (2). Teenagers aged 15–19 years represent about 73% of pediatric MM cases; patients aged 10–14 years of age represent about 17%, while those aged 5–9 years and 1–4 old represent 6 and 4%, respectively (3). Overall, MM incidence in pediatric patients ranges from 1.1 per million in children younger than 5 years to 10.4 per million in those aged 15–19 years in the United States (4). However, data about trends in subjects aged less than 20 years are poor and contrasting (5, 6). In 2011, a literature review reported an incidence increase of 1–4% per year in the pediatric population (5). Conversely, Campbell et al. observed a decreased incidence in teenagers from 2004 to 2010 in the United States (6).

These data highlights how our understanding of pediatric MM is limited because clinical studies rarely involve children and adolescents. In addition, MM diagnosis in children is challenging, as it exhibits a wide range of clinical presentations (7). Clinical surveys have reported that MM in younger children might be amelanotic, uniformly pigmented, bleeding, thicker, and more frequently associated with lymph node metastasis compared to MM in adult patients, and thus displays a different biological behavior (8, 9).

A correct diagnosis is mandatory, as the patient’s management and the correct therapy are directly dependent on diagnosis. Indeed, the therapeutic options for MM include not only surgery, but also targeted therapy using BRAF and MET inhibitors and immunotherapy. Moreover, a better understanding of the MM molecular landscape has led to the identification of new prognostic biomarkers (ALK, NTRK, MYC, C-KIT, and others) and will allow new targets for therapy in the near future (10). The diagnosis of melanocytic lesions is one of the most difficult aspects of dermatology and pathology. The development of dermoscopy in the last decades has improved the recognition of atypical lesions that need to be excised. However, the diagnosis still relies on histological examination, and the differential diagnosis in pediatric patients mainly includes Spitz nevus, atypical Spitz tumors, and Spitz melanoma. Histological diagnosis of melanocytic proliferations is certainly a challenge, as it mainly relies on morphological findings, which are almost partially subjective and requires trained pathologists with specific expertise (11). Recently, immunohistochemical and molecular biomarkers have been applied to the differential diagnosis, and have improved the diagnostic specificity.

Immunohistochemistry (IHC) is one of the most used techniques in pathology laboratories, as it is inexpensive, automatized, and can precisely evaluate the cellular population expressing a specific protein. Several immunohistochemical markers are tested on melanocytic neoplasms in everyday practice, mainly including HMB45, p16, and Ki67. Nevertheless, IHC plays an ancillary role in the diagnosis of melanocytic neoplasms in pediatric patients, and no immunohistochemical marker is entirely specific in differentiating benign from malignant neoplasms.

PRAME (PReferentially expressed Antigen in MElanoma) is a tumor-associated antigen recently identified in some neoplasms, including myxoid liposarcoma, synovial sarcoma, and MM (12). Current data suggest that PRAME is expressed by MM cells, but not by benign melanocytic neoplasms, and consequently it may be applied in the differential diagnosis of challenging melanocytic lesions. However, data about the expression of PRAME by melanocytic lesions in pediatric patients are limited. To fill the gap in this field, our study aims were twofold: first, to provide a description of cases presenting with suspected pigmented skin lesions and clinical findings of atypical melanocytic neoplasms including MM based on the experience of three hospital centers, and second, to evaluate the expression of PRAME in the subset of atypical spitzoid neoplasms in children.

## Materials and Methods

### Patient Cohort

We retrospectively included clinical and histopathological data of children and adolescents referred to participating institutions for pigmented skin lesions suspected of melanoma. Three centers participated in the study: Santobono Hospital (Naples, Italy), the Pediatric Surgery Unit of University of Campania Luigi Vanvitelli (Naples, Italy), and the Pediatric, Adolescents and Young Adults Surgery Division of University of Pisa (Pisa, Italy). From databases containing data of patients subjected to excisional biopsy at these three centers, we selected patients satisfying the following criteria for inclusion in the study: 1) subjects referred to the participating centers from 2006 to 2020; 2) age ≤16 years; and 3) availability of demographic, clinical, surgical, and histopathological results. Data regarding benign pigmented skin lesions were obtained as a control group.

The present study was retrospectively conducted using archival biological samples. The diagnoses had already been rendered in all included cases. Approval by the participating institutions ethical review boards was collected.

At diagnosis, each patient received a baseline evaluation, which included medical history assessment and physical examination. Demographic and clinic characteristics included: sex, age, anatomical site of onset, signs of bleeding, itching, growth speed, and shape/color changes. Surgical characteristics recorded were removal of sentinel lymph node and sentinel lymph node state.

Through a telephone history we also obtained data about the presence of possible risk factors, such as clear skin phenotype, UV exposure levels, familiarity for skin melanoma in first degree relatives, number and presence of congenital nevi, dysplastic nevus syndrome, immunodeficiency status, and residence in polluted areas in patients that were diagnosed with either *in situ* or invasive MM.

This study was reviewed and approved by the ethics committee of University of Campania Luigi Vanvitelli (Naples, Italy).

### Morphological Evaluation

Histological slides of all cases were reviewed by two experienced pathologists trained in melanocytic pathology. Histological studies were performed when necessary for diagnostic purposes. The histological review included immunohistochemical slides, when available. In some cases, further immunohistochemical markers were tested for diagnostic purposes, including HMB45 and p16. We applied diagnostic criteria defined in the most recent WHO classification of skin tumors (13).

### PRAME Immunohistochemistry

Inclusion criteria for PRAME IHC included: 1) spitzoid morphology; and 2) availability of archived residual biomaterial in paraffin blocks. Immunocytochemistry was performed on 5-micron thick sections cut from formalin-fixed and paraffin-embedded (FFPE) tissue blocks. A commercially available anti-PRAME monoclonal antibody (dilution 1:200; EPR20330, Abcam, Cambridge, United Kingdom) was used on the Ventana Bench Mark Ultra System, (Ventana, Oro Valley, USA) autostainer platform, according to the manufacturer’s instructions. The staining of PRAME IHC was recorded as the percentage of immunoreactive tumor cells with nuclear labeling per total number of tumor cells. A positive control was added to each slide, consisting in a PRAME-positive MM. The non-melanocytic tissue in the slide was considered the negative control.

The immunohistochemical slides were interpreted by two experienced dermatopathologists, evaluating both the intensity of the staining and the percentage of stained neoplastic cells on the total number of neoplastic cells. In cases where a consensus was not obtained, it was achieved through review by a third experienced pathologist. Intensity of the staining was graded as follows: score 1+: slight positivity; score 2+: moderate positivity; score 3+: intense positivity. The percentage of the positive cells was recorded, as well as the location of positive cells in the setting of the lesion (junctional *versus* intradermal).

## Results

### Clinical and Pathological Findings

**Figure 1:**
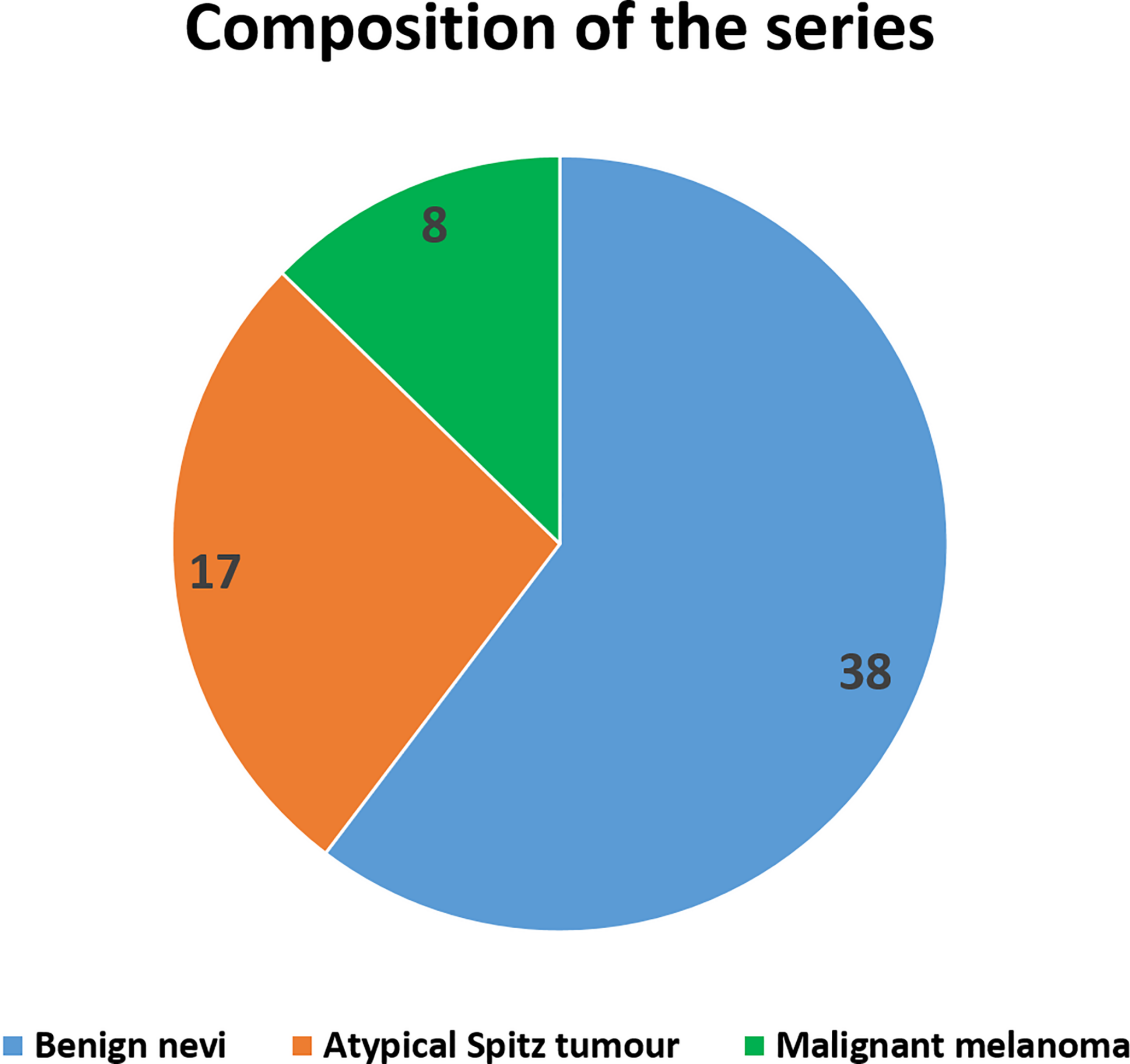
Number of patients included in the study according to skin lesion type.

We evaluated a total of 63 lesions in 63 subjects. Eight of 63 lesions were diagnosed as MM, 17 as atypical Spitz tumor (AST), and 38 as benign nevi (**Figure 1**). Overall, 52% of subjects were males, and the mean age was 6.1 ± 3.3 years. MM lesions were more frequently located in the lower and upper limbs, whereas benign lesions were equally distributed between lower limbs and trunk (see **Table 1**). With regards to clinical characteristics, none of the benign lesions were associated with signs of bleeding and/or itching. One patient exhibited recent shape, dimensions, and color changes of the pre-existing lesion with asymmetry. Moreover, two children presented an increased in size of the lesion. All lesions were diagnosed with cellular dysplasia on histopathologic examination. The remaining benign pigmented lesions underwent surgical excision because of recent fast growth and/or color changes on dermatologic consultation.

**Table 1:** Clinical and demographic characteristics of the pediatric cohort according to lesion type.

Feature	Melanoma (n = 8)	Atypical Spitz Tumor (n = 17)	Benign Pigmented Skin Lesions (n = 38)
Sex			
Male	4	9	20
Signs/Symptoms			
Fast growth	5	17	28
Color changes	3	0	10
Asymmetry	1	0	1
Bleeding	0	0	0
Itching	0	0	0
Site of onset			
Trunk	2	2	12
Upper limb	4	4	7
Lower limb	3	11	12
Head/Neck	0	0	7

With regards to MM lesions, the majority did not arise from a pre-existing nevus and in four cases a rapid growth was reported. No cases of familial melanoma syndrome were observed. One melanoma developed from a congenital nevus and it presented with a rapid change in shape and color. No signs of itching and bleeding were reported.

In this group, a 5-year follow-up was carried out with a survival rate of 100% and neither relapses nor the appearance of metastases occurred. Only one patient underwent an additional surgical excision of a benign skin lesion.

### PRAME Immunohistochemistry

PRAME immunohistochemistry was performed on 38 melanocytic neoplasms with spitzoid features, including 19 Spitz nevi, 17 ASTs, and 2 MMs. Six cases diagnosed as MM were not included in the immunohistochemical evaluation, as no residual bioptic material was available in paraffin blocks after the histological and molecular evaluations performed for diagnostic purposes.

Overall, the mean age of the tested population was 7 years, ranging from 1 to 16 years. For 20 of the 38 (52.6%) cases, lesions were located on the lower limbs, while in 7 (18.4%) cases lesions were located on the trunk, in 6 (15.8%) cases on the upper limbs, and 5 (13.2%) cases on the head and neck region.

Concerning the 19 cases diagnosed as Spitz nevi, the patients ranged in age from 1 to 10 years (mean age: 5.1 years). The lesions were located on the lower limbs in 7 patients (36.8%), on head and neck in 5 (26.3%), on the trunk in 4 (22.2%), and on the upper limbs in 3 (15.8%) patients. One of these patients was diagnosed with a desmoplastic Spitz nevus, with the lesion located on the dorsal trunk of the 8-year-old child (**Table 2**).

**Table 2:** Clinical features of Spitz nevus and atypical Spitz tumor lesions subjected to PRAME immunohistochemistry testing.

Lesion	SN	AST
**Cases**	19	17
**Age (mean age, range)**	5.1; 1–10	7.; 2–13
**Location (N, %)**		
Head and neck	5 (26.3%)	0 (0%)
Trunk	4 (22.2%)	2 (11.8%)
Upper limbs	3 (15.8%)	3 (17.6%)
Lower limbs	7 (36.8%)	12 (70.6%)

SN, spitz nevi; AST, atypical Spitz tumors.

Regarding the 17 patients diagnosed as ASTs, ages ranged from 2 to 13 years (mean age: 7.2 years). Twelve of 17 (70.6%) patients presented lesions on the lower limbs, while in 3 (17.6%) and 2 (11.8%) lesions were located on the upper limbs and the trunk, respectively. The two cases diagnosed as MM presented lesions on the right foot of a 4-year-old child and on the dorsal trunk of a 10-year-old child (**Table 2**).

**Figure 2:**
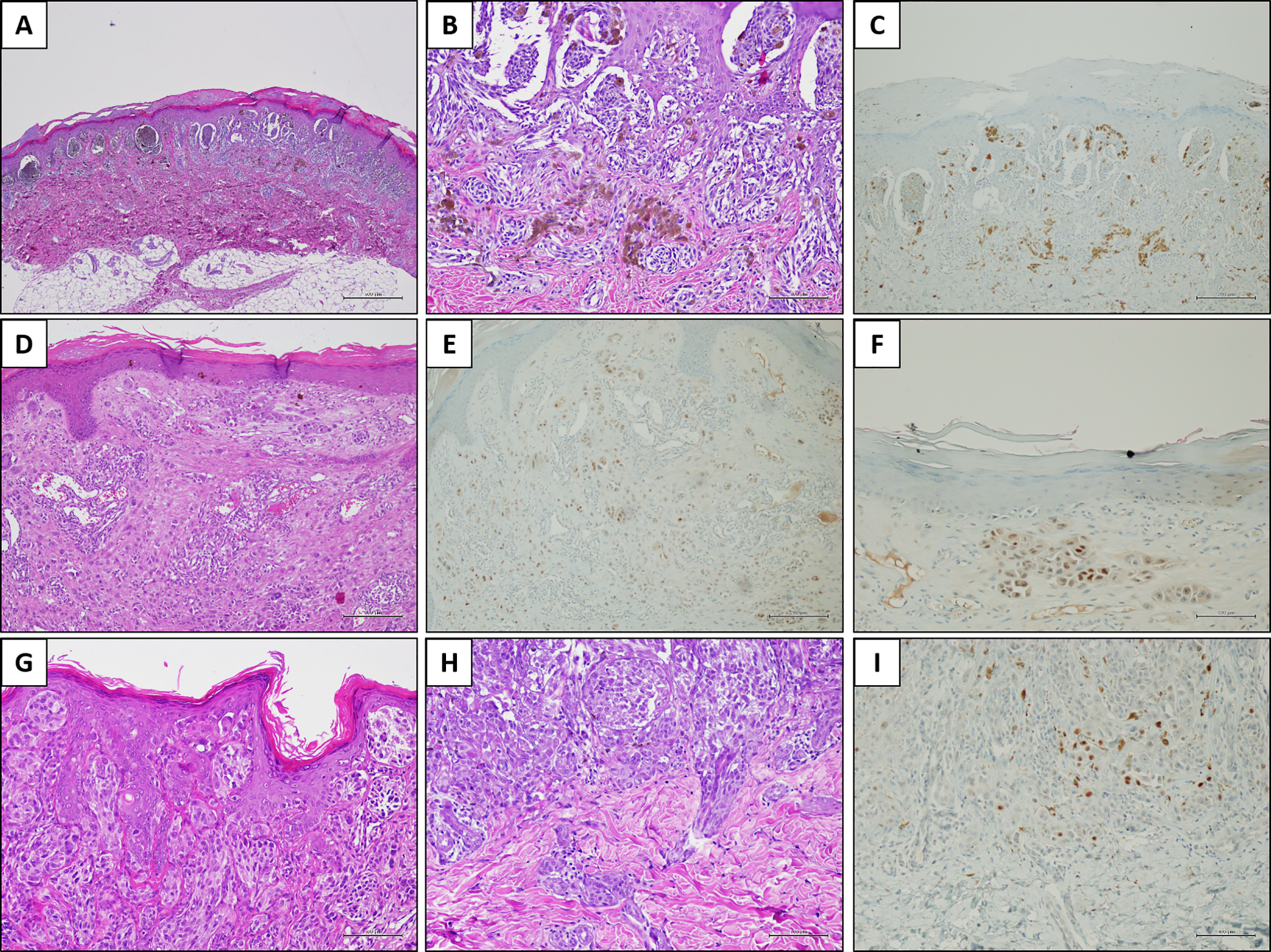
PRAME immunostaining in three explicative lesions. Case 1 (Spitz nevus): a melanocytic lesion located on the right foot of an 8-year-old child. Histologically, the neoplasm was characterized by large junctional nests with peripheral clefting [**(A)** H&E, original magnification 40×]. Some junctional nests are confluent; smaller nests are present in the dermis, in addition to melanophages [**(B)** H&E, original magnification 200×]. PRAME immunostaining was negative [**(C)** immunostaining, original magnification 100×]. Case 2 (Atypical Spitz tumor): a melanocytic lesion located on the leg of an 8-year-old child. In this field, the melanocytic population is arranged in single epithelioid cells and small nests, located in the dermis [**(D)** H&E, original magnification 100×]. Overall, PRAME immunostaining was positive in about 25% of the melanocytic population [**(E)** immunostaining, original magnification 100×] with a moderate (score 2+) intensity [**(F)** immunostaining, original magnification 200×]. Case 3 (Spitz nevus): a melanocytic lesion located on the face of a 3-year-old child. Histologically, the junctional component was organized in confluent nests and constituted by epithelioid and spindle cells, in the context of a hyperplastic epidermis [**(G)** H&E, original magnification 200×]. The dermal component was organized in smaller nests, and peri-adnexal spread was present [**(H)** H&E, original magnification 200×]. PRAME immunostaining showed slight positivity (score 1+) in a few cells, corresponding to the 2% of the melanocytic population [**(I)** immunostaining, original magnification 200×].

Overall, PRAME immunohistochemistry was negative in 33 of 38 (86.8%) cases. Two cases diagnosed with MM tested negative. Five of 38 (13.2%) cases showed some PRAME positivity. PRAME immunohistochemistry testing was positive in 25% of the neoplastic cells in a case of AST arising at the lower limb of an 8-year-old child. The intensity of the staining resulted in a score 2+, and the positive cells included both junctional and intradermal cells. The remaining four positive cases included two ASTs and two SN. In these cases, the percentage of positive cells ranged from 1 to 5%, and the intensity of the staining yielded a score of 1+. The positive cells were junctional in three cases and intradermal in one case (an AST located at the lower limb of a 2-year-old child) (**Figure 2**). The clinical and pathological features of the positive cases are listed in **Table 3**.

**Table 3:** Clinical and pathological features of PRAME-positive cases.

N.	Diagnosis	Location	Age (y)	% Positivity	Score	Location
1	AST	Lower limb	8	25	2+	Junctional and dermal
2	SN	H/N	3	2	1+	Junctional
3	AST	Upper limb	7	5	1+	Junctional
4	SN	Lower limb	2	1	1+	Junctional
5	AST	Lower limb	2	1	1+	Dermal

## Discussion

Although MM is relatively rare, it is the most common skin cancer in pediatric age. The estimated incidence in children under 10 years of age is 1.8 cases for 1 million in the United States (14). MM incidence increases during puberty, with a rate of 14 and 23 cases per million in adolescent males and females, respectively (15). Consequently, MM may be considered a rare tumor in pediatric patients, but the same cannot be said for adolescents. Although MM is a significant problem even in this population, clinical data are insufficient. Moreover, the differential diagnosis of melanocytic neoplasms remains a challenge, mainly in the setting of spitzoid lesions.

Herein, we analyzed a series of melanocytic lesions, and tested the expression of PRAME in a subset of cases. The first part of our study assessed the demographic and clinical presentation of suspected pigmented skin lesions. The ratio of pigmented lesions was equivalent between sexes and the most frequent site of onset was the limbs. The sex distribution of lesions was similar to that reported in previous case series including subjects of similar ages as our study (16). Conversely, in adolescents and youths, females were more frequently diagnosed with MM (17).

In our cohort, we more frequently observed malignant lesions in the lower and upper limbs. This finding is consistent with the data described by Dean et al. who reported the same body distribution for melanoma (16). This trend could be explained, as indicated by Strouse et al. (18), by the greater exposure of the upper and lower limbs to environmental disruptors and/or sunbathing. The latter is considered a risk factor also in adults, as well as phenotypic traits including red hair, blue eyes, and poor tanning ability (19). In addition, if we consider body surface distribution in children compared to adults, in pediatric subjects there is a relative higher prevalence on the upper and lower extremities over trunk surfaces. The common risk factors reported for pediatric melanoma, as well as giant melanocytic nevi, xeroderma pigmentosum, and neurocutaneous melanosis (19) were not detected in our cohort. Moreover, it has been reported in scientific literature that germline variants, such as *MC1R*, *CDKN2A*, and *p16* gene variants are also associated with increased risk of melanoma (19).

Excisional biopsy is mandatory in cases of melanocytic lesions with atypical features in pediatric patients, and the diagnosis relies on histological examination. In this setting, the histological diagnosis of spitzoid neoplasms is one of the most difficult issues in dermatopathology. Despite a better understanding of the molecular biology underlying these neoplasms, the differential diagnosis between benign lesions and malignant lesions is still difficult, and largely based on qualitative and albeit, partially subjective findings (11). PRAME has recently emerged as a novel immunohistochemical marker able to distinguish benign from malignant melanocytic proliferations (20). However, the value of PRAME in the setting of differential diagnosis of spitzoid melanocytic neoplasms in pediatric patients is not well defined. We performed PRAME immunohistochemistry on a series of 38 spitzoid melanocytic neoplasms, including 19 Spitz nevi, 17 ASTs, and 2 MMs. Overall, PRAME was negative in 33 of 38 (86.8%) cases, including three ASTs and two SN. Notably, the two cases diagnosed as MM tested negative as well. In five cases, including three ASTs and two SN, some PRAME positivity was observed. In particular, 25% of both junctional and intradermal neoplastic cells showed a score 2+ staining in an AST located at the lower limb of an 8-year-old child. In the remaining four cases, only a few cells resulted slightly positive (score 1+), ranging between 1 and 5% of the melanocytic population. To more decisively evaluate these results, it is mandatory to define a cut-off value to be applied for positive cases. PRAME stains mainly in the nucleus and consequently the results are of high quality and are easily interpretable in all cases, despite the amount of melanin pigment. In our experience, PRAME staining is diffusely positive, in most neoplastic cells, and in both junctional and intradermal cells, in cases morphologically diagnosed as MM. Nonetheless, we are accustomed to defining negative cases with only few positive cells. Our experience matches observations reported by other studies. Lezcano et al. recently examined the immunohistochemical expression of PRAME in a heterogenous series of 400 melanocytic lesions. The Authors considered PRAME positivity significant when observed in ≥76% of neoplastic cells (20). Similarly, Raghavan et al. defined positive cases showing PRAME staining in at least 60% of the cells (21). Based on these data, in our series the positivity observed in the five cases does not appear to be significant, and we considered all cases tested as negative. Nevertheless, we might speculate that the data reported in literature relied on a higher cut-off value of cellular staining to define PRAME positivity.

Differential diagnosis in the setting of spitzoid melanocytic lesions is challenging, and ancillary tests may be useful. In this setting, PRAME immunohistochemistry is emerging as a novel immunohistochemical test that has been recently introduced in the routine diagnostic work-up of dermatopathologists. When faced with the diagnosis of a melanocytic lesion, a basic immunohistochemistry panel may include HMB45, p16, Ki67, and PRAME expression. However, data regarding the diagnostic value of PRAME in the setting of the spitzoid melanocytic lesions in pediatric patients are missing. In the single paper available in the literature, Raghavan et al. evaluated the expression of PRAME in a series of atypical melanocytic lesions, including 35 spitzoid neoplasms (20 SN, 13 ASTs, and 2 MMs). The authors found that PRAME was expressed in 7.7% of ASTs and in 4% of SNs (21). The study evaluated only two cases of MM, and PRAME expression was in one case (21). However, the series did not consider pediatric patients. In this study, we evaluated PRAME expression specifically focusing on spitzoid lesions in pediatric patients. In our series, PRAME tested negative in all cases. Although some cells showed PRAME expression in five lesions, its expression was focal (25% of the cells in one case and ≤5% in the remaining four cases) and did not reach the cut-off value for positivity. We can conclude that PRAME is not expressed in SN and ASTs in pediatric patients, and therefore it is not useful for the differential diagnosis of SN and AST in this clinical setting. Conversely, we tested only two MMs, and therefore no significant information could be obtained from our series relative to the expression of PRAME in MM lesions in pediatric patients.

In conclusion, in our case series we observed that pediatric MM equally affects young boys and girls, and that the limbs are the most common site of onset. These findings highlight the different clinical behavior of MM in children compared to adults. In addition, we tested PRAME expression in a series of 38 spitzoid melanocytic lesions in pediatric patients. Although PRAME is an emerging IHC marker for the characterization of melanocytic lesions in adults, data regarding its utility in the diagnosis of spitzoid lesions in pediatric patients are lacking. Herein, we demonstrated that PRAME is not expressed in either SN or ASTs in this clinical setting; thus PRAME positivity may be considered an element useful for the differential diagnosis of MM. However, there are insufficient data in pediatric populations about PRAME expression in MM with spitzoid morphology, as only two cases have been reported by a previous study, of which only one case resulted positive. Considering the paucity of clinical and histopathological data in pediatric cohorts, additional studies should be conducted in this field with the aim of identifying predictors of malignant forms.

## Data Availability Statement

The raw data supporting the conclusions of this article will be made available by the authors, without undue reservation.

## Ethics Statement

Ethical review and approval were not required for the study on human participants in accordance with the local legislation and institutional requirements. The patients/participants provided their written informed consent to participate in this study.

## Author Contributions

AR, RF, and GD’A revised the histological and immunohistochemical slides and contributed to design the study. ME and GU contributed to design the study, contributed to write the manuscript and data analysis. VD’O, GD, LT, CS, and SS provided the clinical data and biological material, contributed to the analysis of the data. AP contributed to design the study and reviewed the manuscript. All authors contributed to the article and approved the submitted version.

## Conflict of Interest

The authors declare that the research was conducted in the absence of any commercial or financial relationships that could be construed as a potential conflict of interest.
